# Snapping Pes Syndrome Caused by the Gracilis Tendon: Successful Selective Surgery with Specific Diagnosis by Ultrasonography

**DOI:** 10.1155/2020/1783813

**Published:** 2020-02-28

**Authors:** Manabu Akagawa, Yoshiaki Kimura, Hidetomo Saito, Hiroaki Kijima, Kimio Saito, Toyohito Segawa, Ikuko Wakabayashi, Takeshi Kashiwagura, Naohisa Miyakoshi, Yoichi Shimada

**Affiliations:** ^1^Department of Orthopedic Surgery, Akita City Hospital, 4-30 Kawamoto-Matsuokacho, Akita 010-0933, Japan; ^2^Department of Orthopedic Surgery, Akita University Graduate School of Medicine, 1-1-1 Hondo, Akita 010-8543, Japan; ^3^Department of Orthopedic Surgery, Joto Sports Orthopaedic Clinic, 7-1-3 Nakadori, Akita 010-0001, Japan

## Abstract

We report the case of painful snapping pes syndrome caused by the gracilis tendon. A 26-year-old man presented with acute right knee pain and restricted extension. Although snapping could not be reproduced due to severe pain, the snapping of the gracilis tendon could be specifically diagnosed using ultrasonography and lidocaine injection. Because of the failure of conservative treatment, surgery was performed. The distal attachment of the gracilis tendon was released, and the symptom disappeared quickly. There was no recurrence at the 10-month follow-up.

## 1. Introduction

Snapping of the pes anserinus tendon is uncommon, but there are several case reports [1–4]. However, identifying the specific structure responsible for the snapping remains difficult, because there are multiple causes in the affected area, and sometimes, even normal structures cause the snapping. Those without an obvious abnormality are called snapping pes syndrome (SPS) [5].

Ultrasonography (US) is a useful diagnostic tool [5–8], because snapping occurs during joint motion, and only US can visualize the snapping dynamically. However, despite evaluating snapping dynamically with US, both semitendinosus and gracilis tendons were resected or harvested without clarifying which is the true cause in almost all reports.

An SPS case that was specifically diagnosed with US and lidocaine injection and treated with selective, minimally invasive surgery is presented.

## 2. Case Presentation

A 26-year-old man visited a nearby hospital with the chief complaint of acute right knee pain and restricted extension without any apparent cause. Intra-articular injection and drug therapy did not improve the symptom. Therefore, he came to our department the following day. The same symptoms had occurred several times since the age of 17 years, and each time, he reduced it by himself, but this time he could not. He did not have any specific sports or daily activities which cause snapping.

Physical examination showed no redness, swelling, or ballottement in the right knee; range of motion restricted to extension -20° and flexion 120° due to severe pain; and tenderness in the medial joint space and proximal part of the medial tibial condyle.

**Figure 1:**
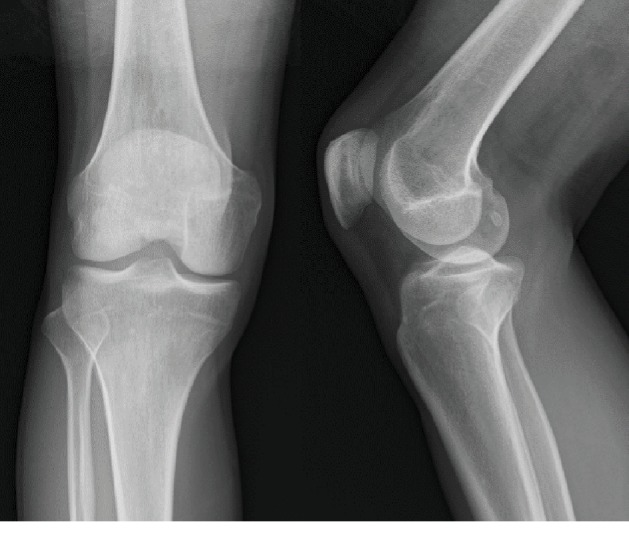
Preoperative radiographs of the knee. Radiographs showed no abnormalities.

**Figure 2:**
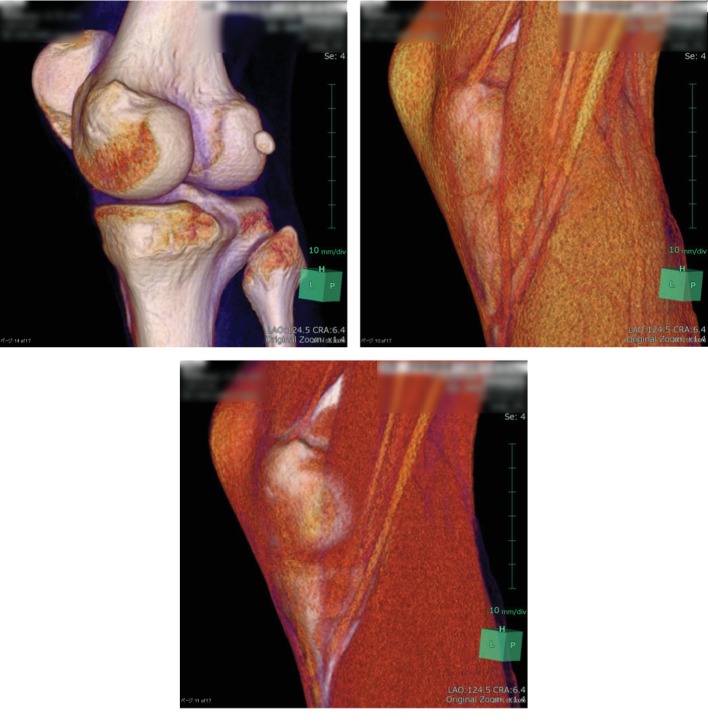
Three-dimensional reconstructed computed tomography images. There was no anatomical abnormalities in the bone and pes anserinus.

**Figure 3:**
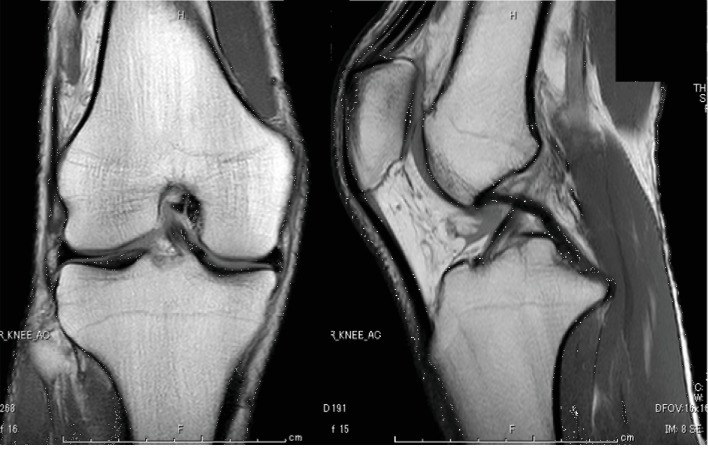
Magnetic resonance imaging of the knee. There was no evidence of intra- or extra-articular abnormalities.

Radiographs, computed tomography, and magnetic resonance imaging showed no evidence of intra- or extra-articular abnormalities (Figures 1-3). However, US showed snapping of the gracilis tendon. It was not possible to reproduce the snapping symptom due to pain, but when each tendon of the pes anserinus was slid manually under US, the pain was reproduced only in the gracilis tendon ([Supplementary-material supplementary-material-1]). Thus, lidocaine injection was performed under US guidance. Since selective injection of the local anaesthetic to only the gracilis tendon allowed the patient to fully extend the knee without pain, SPS was diagnosed. However, the injection's effect disappeared in half a day, and severe pain reappeared. Therefore, surgery was performed.

**Figure 4:**
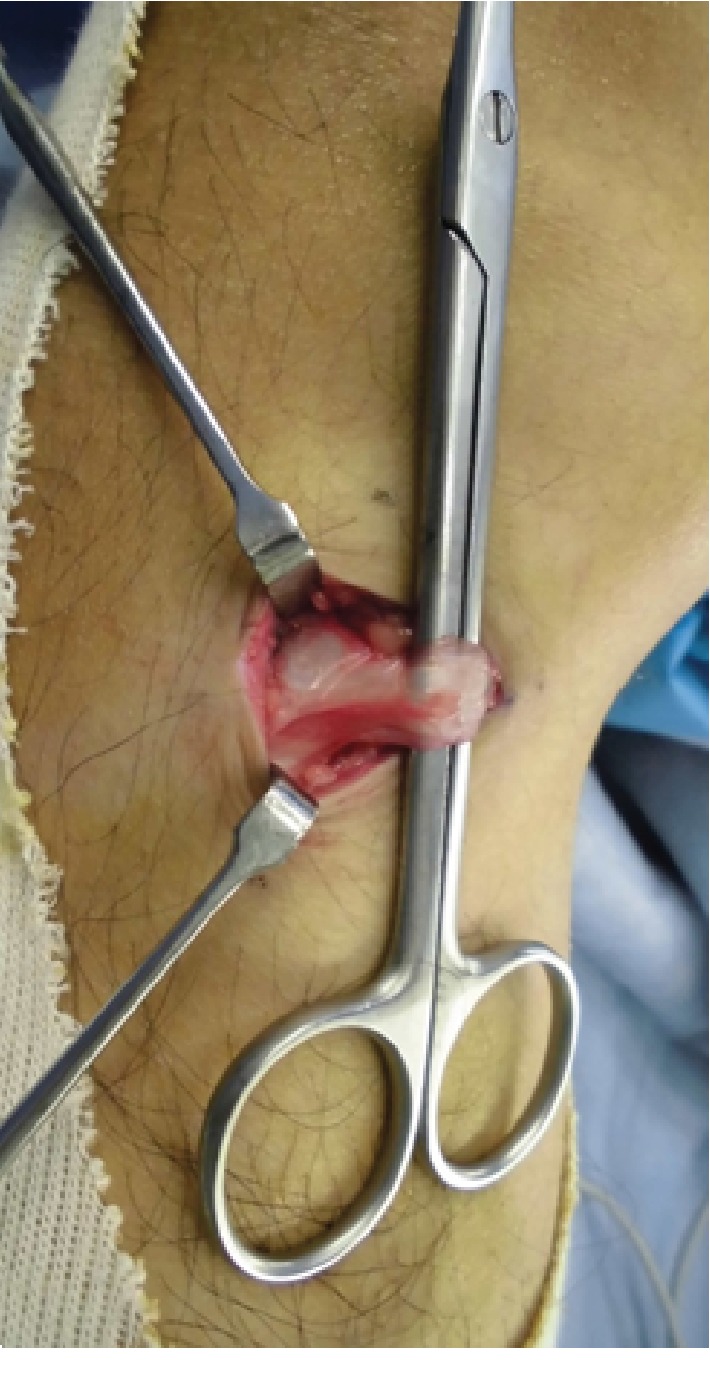
Surgically identified gracilis tendon. In the operation, the gracilis tendon was identified, and only the distal attachment was released.

Under general anaesthesia, gracilis tendon snapping was confirmed visually ([Supplementary-material supplementary-material-1]). The distal attachment of the gracilis tendon was released (Figure 4), and the snapping disappeared. Immediately after the surgery, the patient could walk and fully extend the knee without pain. There was no recurrence at the 10-month follow-up. The Lysholm score improved from 35 before surgery to 100 at 10 months after surgery.

## 3. Discussion

The results of this case highlight two important clinical issues. First, it was possible to accurately diagnose the specific structure responsible with dynamic US and lidocaine injection.

A past report suggested a reasonable hypothesis for the cause of SPS [9]. They hypothesized that the combination of repetitive overloading and abnormalities in the accessory band of the pes anserinus tendon, which stabilizes the tendons, causes SPS. Another review paper also suspected the deficiency of the accessory band as a cause of SPS [10]. However, anatomical studies showed that both gracilis and semitendinosus have several accessory bands [11] with high variability [12]. Additionally, although US is useful to visualize and diagnose the snapping phenomenon, it is difficult to draw out the accessory band itself. These anatomical features make it more difficult to specifically diagnose the responsible structure of SPS in the clinical situation. In the present case, although we could not reveal anatomical abnormalities of the accessory band, lidocaine injection was used in combination with US, and the gracilis was finally diagnosed as the main cause of the snapping. With this specific diagnosis, it was possible to reduce unnecessary tendon detachment and avoid functional loss.

Second, releasing only the distal attachment of the tendon solved the symptom of SPS. Treatment for SPS usually consists of tenotomy with or without partial resection of both gracilis and semitendinosus tendons [5, 7, 8, 13] (Table 1). In these reports, the unnecessary tendon may have been resected because the responsible structure was not specifically identified. Additionally, a recent report suggested that releasing only the distal attachment suffices [14]. Therefore, resecting both gracilis and semitendinosus tendons is highly invasive and may result in functional loss. In the present case, we identified the gracilis tendon as the responsible structure and released only the distal attachment of it. The difference in hamstring muscle strength and in the long-term results between resecting and releasing of the distal attachment is unclear, but surgical invasiveness may be less with releasing of the distal attachment. To our knowledge, this is the first report that specifically diagnosed the responsible structure of SPS and that was treated with selective, minimally invasive surgery.

## 4. Conclusion

A patient with SPS caused by the gracilis tendon was specifically diagnosed by US and lidocaine injection. Selective, minimally invasive surgery resulted in resolution of the patient's symptom with no recurrence at the 10-month follow-up.

## Figures and Tables

**Table 1:** Diagnostic measures and surgical procedures of previous reports.

Author	Diagnostic measures	Surgical procedure
S.R. Bollen et al.	US	Resected both ST and G
C.E. Rainey et al.	US	Resected both ST and G
B. de la Hera Cremades et al.	US	Resected both ST and G
S.A. Shapiro et al.	US	Resected both ST and G
Present case	US and lidocaine injection	Released only the distal attachment of G

US: ultrasonography; ST: semitendinosus tendon; G: gracilis tendon.

## Supplementary Materials

Supplementary 1 Movie 1: confirmation of snapping phenomenon on ultrasonography. When the gracilis tendon was manually slid, the snapping phenomenon was reproduced.

Supplementary 2 Movie 2: snapping phenomenon under general anaesthesia. The snapping phenomenon was visually confirmed under general anaesthesia.

## References

[1] Bae D. K., Kwon O. S. (1997). Snapping knee caused by the gracilis and semitendinosus tendon. A case report. *Bulletin/Hospital for Joint Diseases*.

[2] Karataglis D., Papadopoulos P., Fotiadou A., Christodoulou A. G. (2008). Snapping knee syndrome in an athlete caused by the semitendinosus and gracilis tendons. A case report. *The Knee*.

[3] Seino D., Nakayama H., Imamura F., Moro-oka T., Yoshiya S. (2014). Snapping knee caused by the gracilis tendon: a case report with an anatomical study. *Asia-Pacific Journal of Sports Medicine, Arthroscopy, Rehabilitation and Technology*.

[4] von Dercks N., Theopold J. D., Marquass B., Josten C., Hepp P. (2016). Snapping knee syndrome caused by semitendinosus and semimembranosus tendons. A case report. *The Knee*.

[5] Bollen S. R., Arvinte D. (2008). Snapping pes syndrome: a report of four cases. *Journal of Bone and Joint Surgery British Volume*.

[6] Hung C. Y., Chang K. V., Lam S. (2018). Dynamic sonography for snapping knee syndrome caused by the gracilis tendon. *Journal of Ultrasound in Medicine*.

[7] Rainey C. E., Taysom D. A., Rosenthal M. D. (2014). Snapping pes anserine syndrome. *Journal of Orthopaedic & Sports Physical Therapy*.

[8] Shapiro S. A., Hernandez L. O., Montero D. P. (2017). Snapping pes anserinus and the diagnostic utility of dynamic ultrasound. *Journal of Clinical Imaging Science*.

[9] Lyu S. R., Wu J. J. (1989). Snapping syndrome caused by the semitendinosus tendon. A case report. *The Journal of Bone and Joint Surgery American Volume*.

[10] Marchand A. J., Proisy M., Ropars M., Cohen M., Duvauferrier R., Guillin R. (2012). Snapping knee: imaging findings with an emphasis on dynamic sonography. *American Journal of Roentgenology*.

[11] Yasin M. N., Charalambous C. P., Mills S. P., Phaltankar P. M. (2010). Accessory bands of the hamstring tendons: a clinical anatomical study. *Clinical Anatomy*.

[12] Candal-Couto J. J., Deehan D. J. (2003). The accessory bands of gracilis and semitendinosus: an anatomical study. *The Knee*.

[13] de la Hera Cremades B., Escribano Rueda L., Lara Rubio A. (2017). Snapping knee caused by the thickening of the medial hamstrings. *Revista Española de Cirugía Ortopédica y Traumatología*.

[14] Geeslin A. G., LaPrade R. F. (2010). Surgical treatment of snapping medial hamstring tendons. *Knee Surgery, Sports Traumatology, Arthroscopy*.

